# Development of a prediction model for mortality and cardiovascular outcomes in older adults taking into account AZGP1

**DOI:** 10.1038/s41598-021-91169-4

**Published:** 2021-06-03

**Authors:** Dörte Huscher, Natalie Ebert, Inga Soerensen-Zender, Nina Mielke, Elke Schaeffner, Roland Schmitt

**Affiliations:** 1 Institute of Public Health, Charité Universitätsmedizin Berlin, Berlin, Germany; 2 Institute of Biometry and Clinical Epidemiology, Berlin Institute of Health, Charité Universitätsmedizin Berlin, Berlin, Germany; 3 Department of Nephrology and Hypertension, Medizinische Hochschule Hannover (MHH), Carl-Neuberg-Str. 1, 30625 Hannover, Germany

**Keywords:** Biomarkers, Cardiology, Risk factors

## Abstract

Zinc-alpha 2-glycoprotein (AZGP1) is a serum protein with postulated functions in metabolism, cancer and cardiovascular disease. We developed new prediction models for mortality or cardiovascular events investigating the predictive potential of serum AZGP1 in a community-based cohort of older adults. We measured AZGP1 (μg/ml) in stored serum samples of 930 individuals of the Berlin Initiative Study, a prospective, population-based cohort of adults aged ≥ 70. We determined the prognostic potential of 20 knowledge-based predictors including AZGP1 for the outcomes of mortality or the composite endpoint of death and cardiovascular events (stroke, myocardial infarction (MI)) using Cox models; their model fit was evaluated with calibration plots, goodness-of-fit tests and c-indices. During median follow-up of 48.3 months, 70 incident strokes, 38 incident MI and 234 deaths occurred. We found no associations or correlations between AZGP1 and other candidate variables. After multivariable Cox regression with backward-selection AZGP1 remained in both models for mortality (HR = 0.44, 95%CI: 0.24–0.80) and for the composite endpoint (HR = 0.43, 95%CI: 0.23–0.82). Within newly built prediction models, we found that increased AZGP1 levels were predictive for lower risk of mortality and the composite endpoint in older adults. AZGP1 as a predictor warrants further validation in older adults.

## Introduction

Zinc-alpha 2-glycoprotein, AZGP1 (often also abbreviated as ZAG) is a secreted 43 kDa protein which is expressed in many epithelial tissues and in adipocytes. AZGP1 circulates at high concentrations in human blood.

Despite a multitude of proposed implications in different diseases, the true role of AZGP1, which shows inverse variations with glomerular filtration rate^1–3^, is still poorly defined. AZGP1 has been suggested as a modifier of metabolic functions, insulin sensitivity, fat mass expansion, blood pressure regulation, cancer progression, inflammatory disease, vitiligo, neurological disease and cardiovascular (CV) disease^4–9^. Experimentally AZGP1 exerts antifibrotic effects in kidney and heart^10^ and indirect clinical evidence indicates that AZGP1 can act as an autocrine/paracrine adipokine to promote lipolysis and improve insulin resistance^4,11–13^. Through these and other effects, AZGP1 might modify the risk for metabolic syndrome, CV disease and mortality^14–17^. However, previous studies have provided partially conflicting findings about the potential role of circulating AZGP1 on CV outcomes and mortality in different populations. While serum AZGP1 correlated with CV disease and mortality in dialysis patients, there was an inverse relationship with atherosclerosis and coronary heart disease in non-dialysis patients^12,18,19^.

CV disease is the leading cause of morbidity and death in individuals over the age of 65. Typically, older adults have a more heterogeneous burden of comorbidities and the pathomechanistic course of CV disease shows more variability compared to younger patients^20,21^. An important factor contributing to this variability are age- and health-dependent changes of renal function^22,23^. Improving risk prediction of CV disease has been difficult in the elderly and existing prediction modeling suboptimal^24^.

The aim of the present study was to build prediction models for death and the composite endpoint of death and cardiovascular events (including stroke and myocardial infarction) in old age. Therefore, we investigated the prognostic potential of several knowledge-based predictors including biomarkers for kidney function and serum AZGP1 levels for these outcomes within the Berlin Initiative Study, a population-based cohort of older adults initiated to study kidney function at older age^25,26^.

## Results

### Cohort characteristics

Baseline characteristics of the study population are presented in Table 1 (stratified by quartiles of log-transformed AZGP1 serum levels. The median duration of follow-up was 48.3 months. Of the 930 individuals analyzed, 53% were female, mean age was 82 years, and mean BMI was 28 kg/m^2^; 63% had a CCI ≥ 8. The mean eGFR_BIS2_ was 52 ml/min/1.72 m^2^, and median albumin-to-creatinine-ratio (ACR) was 11 mg/g. Based on health insurance data, 17% of study participants had experienced a prior MI and 16% a stroke before enrolment into the study. Mean log-transformed AZGP1 was 1.92 μg/ml (Table 1). As the 930 individuals with AZGP1 measurement (64.6%) were a subsample of the total sample (n = 1440) of the second BIS follow-up visit, we compared them to the 510 individuals (35.4%) without AZGP1 measurements. Overall, the two groups exhibited similar characteristics, with the AZGP1 study population being on average 2 years younger, having slightly more smokers, less comorbidities, and a tendency to lower ACR values (Supplementary Table S1).

**Table 1:** Baseline characteristics of total study sample and according to log-transformed AZGP1 levels in older adults.

	Total study sample	log(AZGP1) quartile groups			
		≤ 1.76	> 1.76–1.93	> 1.93–2.07	> 2.07
N (% of cohort)	930 (100)	229 (24.6)	241 (25.9)	218 (23.4)	242 (26.0)
Age (years), mean ± SD	82.3 ± 5.6	81.5 ± 5.2	82.3 ± 5.4	82.9 ± 5.6	82.4 ± 6.0
Female, n (%)	491 (52.8)	121 (52.8)	143 (59.3)	111 (50.9)	116 (47.9)
Smoking ever, n (%)	460 (49.5)	118 (51.5)	103 (42.7)	107 (49.1)	132 (54.5)
Body mass index (kg/m^2^), mean ± SD	27.8 ± 4.4	28.3 ± 4.4	27.7 ± 4.3	27.3 ± 4.3	27.7 ± 4.5
Waist to hip ratio, mean ± SD	0.92 ± 0.08	0.92 ± 0.08	0.92 ± 0.08	0.92 ± 0.08	0.92 ± 0.07
Antihypertensive medication, n (%)	761 (81.9)	183 (79.9)	196 (81.3)	180 (82.6)	202 (83.8)
Diabetes mellitus, n (%)	215/921 (23.3)	52/227 (22.9)	60/237 (25.3)	46/218 (21.1)	57/239 (23.8)
Myocardial infarction^a^, n (%)	157/922 (17.0)	39/227 (17.2)	37/239 (15.5)	32/217 (14.7)	49/239 (20.5)
Stroke^a^, n (%)	149/922 (16.2)	38/227 (16.7)	36/239 (15.1)	35/217 (16.1)	40/239 (16.7)
Heart failure, n (%)	36/927 (3.9)	8/229 (3.5)	8/240 (3.3)	6/217 (2.8)	14/241 (5.8)
Cancer^a^, n (%)	323/922 (35.0)	68/227 (30.0)	82/239 (34.3)	81/217 (37.3)	92/239 (38.5)
Anemia, n (%)	161/922 (17.5)	33/226 (14.6)	34/238 (14.3)	39/218 (17.9)	55/240 (22.9)
Charlson comorbity index^b^, n (%)	921	227	239	217	238
3–4	75 (8.1)	20 (8.8)	18 (7.5)	18 (8.3)	19 (8.0)
5–7	264 (28.7)	70 (30.8)	75 (31.4)	58 (26.7)	61 (25.6)
8–10	279 (30.3)	75 (33.0)	79 (33.1)	64 (29.5)	61 (25.6)
≥ 11	303 (32.9)	62 (27.3)	67 (28.0)	77 (35.5)	97 (40.8)
Systolic blood pressure (mmHg), mean ± SD	143.3 ± 21.6	143.8 ± 20.2	142.2 ± 23.3	144.3 ± 20.3	142.9 ± 22.5
Diastolic blood pressure (mmHg), mean ± SD	79.4 ± 12.9	79.9 ± 11.7	79.3 ± 13.6	79.3 ± 13.0	79.2 ± 13.1
Hemoglobin (g/dl)	13.5 ± 1.4	13.7 ± 1.3	13.6 ± 1.2	13.5 ± 1.3	13.3 ± 1.5
Cholesterol level (mg/dL), mean ± SD	213.7 ± 50.2	211.6 ± 47.2	212.1 ± 46.7	217.8 ± 54.1	213.8 ± 52.8
LDL cholesterol (mg/dL), mean ± SD	123.1 ± 42.3	122.8 ± 40.9	121.0 ± 38.9	126.2 ± 46.1	122.7 ± 43.4
HDL cholesterol (mg/dL), mean ± SD	62.0 ± 19.5	60.4 ± 17.5	64.3 ± 20.3	63.1 ± 18.9	60.1 ± 20.7
CRP (mg/dl), mean ± SD	3.3 ± 5.5	3.3 ± 5.9	2.7 ± 3.3	3.3 ± 5.2	3.9 ± 6.9
AZGP1 (μg/ml), median (min, max)	84.2 (9.7, 347.6)	43.8 (9.7, 57.5)	71.9 (57.7, 84.9)	101.2 (85.2, 117.4)	151.7 (117.6, 347.6)
log AZGP1, mean ± SD (min, max)	1.92 ± 0.23	1.61 ± 0.13 (0.99, 1.759)	1.85 ± 0.05 (1.76, 1.929)	2.00 ± 0.04 (1.93, 2.0697)	2.20 ± 0.10 (2.07, 2.54)
Serum creatinine (mg/dl), mean ± SD	1.04 ± 0.39	0.95 ± 0.31	0.96 ± 0.25	1.03 ± 0.32	1.21 ± 0.54
Cystatin C (mg/l)	1.35 ± 0.45	1.24 ± 0.34	1.27 ± 0.32	1.35 ± 0.40	1.53 ± 0.60
eGFR_BIS2_ (ml/min/1.73 m^2^), mean ± SD	51.6 ± 13.2	55.5 ± 12.4	53.3 ± 12.1	50.9 ± 12.0	46.8 ± 14.5
eGFR _BIS2_ < 60, n (%)	674/926 (72.8)	149/228 (65.4)	169/240 (70.4)	165/218 (75.7)	191/240 (79.6)
eGFR _BIS2_ < 45, n (%)	288/926 (31.1)	48/228 (21.1)	59/240 (24.6)	70/218 (32.1)	111/240 (46.3)
eGFR _BIS2_ < 30, n (%)	58/926 (6.3)	4/228 (1.8)	3/240 (1.3)	13/218 (6.0)	38/240 (15.8)
ACR (mg/g), median (IQR)	11.3 (4.8; 33.9)	10.1 (4.7, 31.3)	9.7 (4.5, 23.7)	11.9 (5.2, 30.8)	16.4 (5.6, 49.9)

*ACR* albumin creatinine ratio, *CRP* c-reactive protein, *eGFR* estimated glomerular filtration rate, *HDL* high-density lipoprotein, *LDL* low-density lipoprotein.

^a^Myocardial infarction, stroke and cancer were derived from insurance claims data based on ICD-10 codes.

^b^Since for the age-adjusted Charlson comorbidity index no established grouping is available, classification was based on survival differences of patients according to the combined age-comorbidity score of the validation paper^40^; the remaining category “ > 7” was split into two further groups of similar size for better distinction.

### Baseline variables according to AZGP1 levels

For most of the baseline variables no distinct pattern such as an increase or decrease with regard to the AZGP1 concentration could be observed (Table 1). No difference in mean AZGP1 levels was seen between women (1.91 ± 0.23) and men (1.93 ± 0.23, p = 0.13). All kidney function measures, however, demonstrated worsening kidney function (an increase in serum creatinine or cystatin C levels or a decline in eGFR) with increasing levels of AZGP1. While we found no differences in CCl between the lower AZGP1 groups, we observed that the highest quartile group was associated with higher CCI categories. As further analysis revealed that individuals in the highest CCl category were also more likely to exhibit signs of kidney impairment (decreased GFR) and kidney damage (albuminuria), we repeated the analysis excluding individuals categorized as CKD with “very high risk” regarding kidney disease prognosis according to the KDIGO guidelines (see Figure “Prognosis of CKD by GFR and albuminuria category” in ^27^). In the analysis excluding individuals with GFR category G3a and worse with persistent albuminuria category A3 (eGFR < 60 ml/min/1.73 m^2^ and ACR > 300 mg/g), GFR category G3b and worse with persistent albuminuria category A2 (eGFR < 45 ml/min/1.73 m^2^ and ACR ≥ 30 mg/g), and GFR category G4 and G5 (eGFR < 30 ml/min/1.73 m^2^) the association of AZGP1 with the highest CCI category vanished (Table 2). Also, AZGP1 correlated with none of the continuous baseline variables (Table 3).

**Table 2:** Indicators of CKD and AZGP1 by CCI categories in the subsample and when excluding individuals without high risk of CKD according to KDIGO guidelines*.

	Charlson comorbidity index, age-corrected				p
	3–4	5–7	8–10	> = 11	
**eGFR**_**BIS2**_** (ml/min/ 1.73 m**^**2**^**), n (%)**					
< 60 < 45	36 (48.6) 4 (5.4)	148 (56.3) 32 (12.2)	224 (80.6) 97 (34.9)	262 (86.8) 155 (51.3)	–
< 30	0	1 (0.4)	15 (5.4)	42 (13.9)	
**ACR (mg/g), n (%)**					
≥ 30 > 300	12 (16.0) 1 (1.3)	50 (19.0) 4 (1.5)	76 (27.2) 13 (4.7)	112 (38.6) 15 (5.2)	–
log(AZGP1), mean ± SD	1.90 ± 0.21	1.89 ± 0.24	1.90 ± 0.24	1.97 ± 0.22	0.020
Excl. individuals with GFR stage G4, G5, G3b with A2 or G3a with A3^a^ (n = 768)					
log(AZGP1), mean ± SD	1.90 ± 0.22	1.88 ± 0.24	1.88 ± 0.23	1.92 ± 0.20	0.45

^a^Prognosis of CKD classified as “very high risk” according to KDIGO guidelines: eGFR < 30 ml/min/1.73 m^2^ or eGFR < 45 ml/min/1.73 m^2^ and ACR >  = 30 mg/g or eGFR < 60 ml/min/1.73 m^2^ and ACR > 300 mg/g^27^.

**Table 3:** Correlation coefficients (CC) of log-transformed AZGP1 levels with demographic parameters and biomarkers.

	Pearson CC	Spearman CC
Age	0.080	0.066
BMI (kg/m^2^)	−0.030	−0.053
Waist-hip ratio	0.046	0.039
CCI	0.149	0.137
Systolic blood pressure (mmHg)	0.003	−0.006
Diastolic blood Pressure (mmHg)	−0.011	−0.020
Hemoglobin (g/dl)	−0.086	−0.084
Serum creatinine (mg/dl)	0.252	0.222
Cystatin C (mg/l)	0.270	0.229
Cholesterol (mg/dl)	0.035	0.031
HDL cholesterol (mg/dl)	−0.003	−0.022
LDL cholesterol (mg/dl)	0.009	0.007
CRP (mg/l)	0.052	0.048
log(CRP)	0.047	0.048
eGFR _BIS2_ (ml/min/1.73 m^2^)	−0.252	−0.233
ACR (mg/g)	0.073	0.105
log(ACR)	0.118	0.107

*ACR* albumin creatinine ratio, *BMI* body mass index, *CCI* Charlson comorbidity index, *CRP* c-reactive protein, *eGFR* estimated glomerular filtration rate, *HDL* high-density lipoprotein, *LDL* low-density lipoprotein, *log* natural logarithm.

### Prediction of death

**Figure 1:**
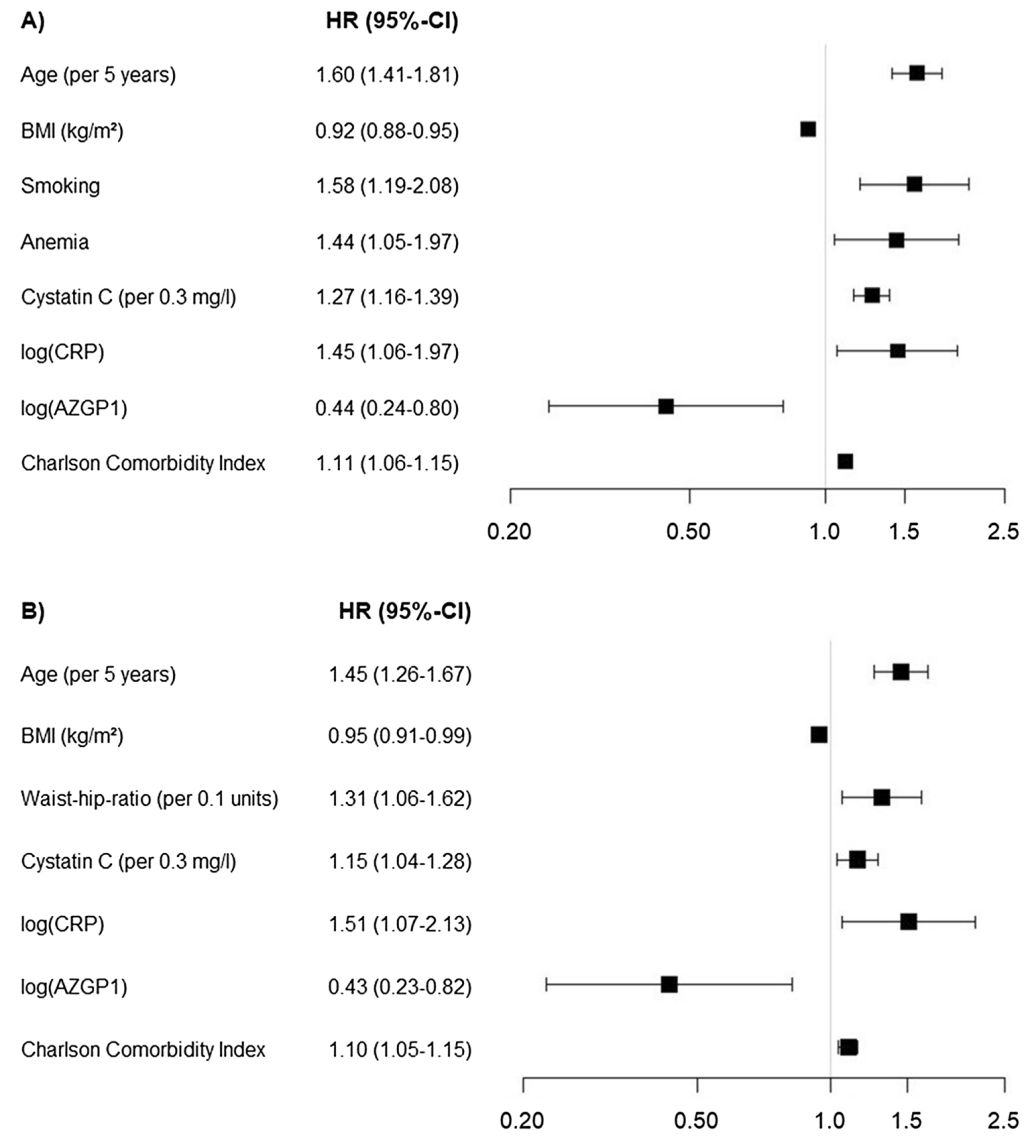
Predictors for **(A)** death and **(B)** composite endpoint (endpoint of myocardial infarction, stroke or death) from the multivariable Cox model with stepwise backward selection. *BMI* body mass index, *log* natural logarithm, *CRP* c-reactive protein, *HR* hazard ratio, *CI* confidence interval. The figure was produced with IBM SPSS Statistics version 25.0 (https://www.ibm.com/de-de/analytics/spss-statistics-software) and R version 4.0.0 (https://www.R-project.org).

**Figure 2:**
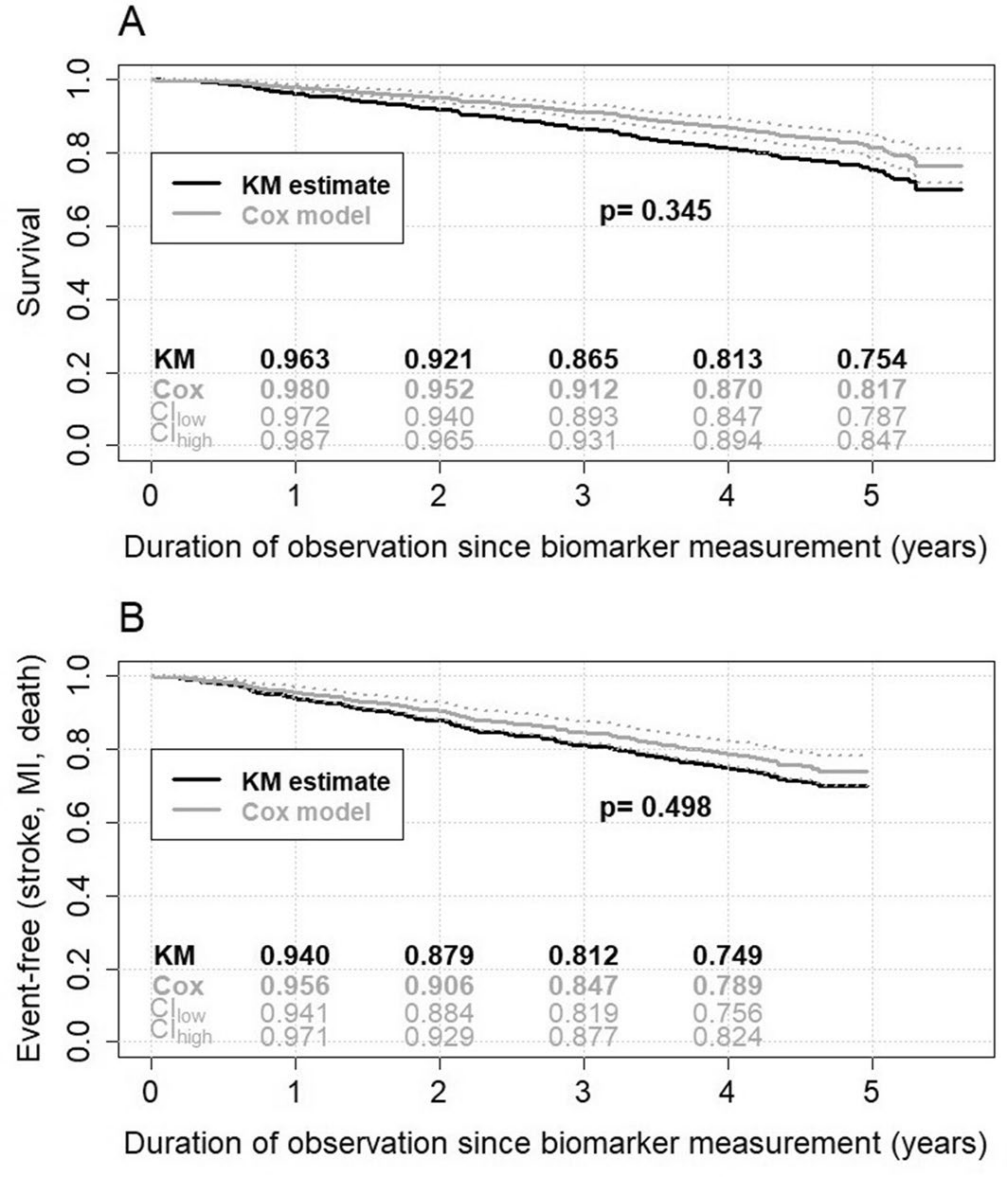
Fit of the multivariable Cox models with 95% confidence interval compared to the estimates from Kaplan–Meier analysis for **(A)** death and **(B)** the composite endpoint. The p-value from a calibration test for the Cox model is shown. *KM* Kaplan–Meier, *CI* confidence interval, *time* duration of observation since biomarker measurement. The figure was produced with IBM SPSS Statistics version 25.0 (https://www.ibm.com/de-de/analytics/spss-statistics-software) and R version 4.0.0 (https://www.R-project.org).

In total, 234 deaths occurred in the subsample, and 218 deaths in the 882 cases with complete candidate variables (Supplementary Fig. S1). There was no difference in survival when comparing the AZGP1 quartiles in Kaplan–Meier analysis (p = 0.110, Supplementary Fig. S2). Out of the 20 candidate variables considered for the prediction of death, from 500 bootstrap-based Cox models with backward selection, AZGP1 was among 8 variables included in > 50% of all models (67.4%, Supplementary Table S2). In multivariable Cox regression analysis of the subsample, the final model incorporated the predictive factors age (HR; 95% CI: 1.60; 1.41–1.81), BMI (0.92; 0.88–0.95), smoking (1.58; 1.20–2.08), anemia (1.44; 1.05–1.97), cystatin C (1.27 per 0.3 mg/l; 1.16–1.39), CRP (1.45 per log(mg/l)-unit; 1.06–1.97), CCI (1.11; 1.06–1.15), and AZGP which demonstrated predictive value (HR per log-unit: 0.44; 0.24–0.8, p = 0.008, Fig. 1A). The c-index of this Cox model was 0.756 (0.755–0.758). The calibration test indicated good model performance (p = 0.345). The model overestimated the observed survival by 2.0–6.5% (Fig. 2A). When using LASSO for variable selection, the same eight variables with similar HR estimates as stated above and eight additional variables were selected (Supplementary Table S2). The corresponding c-index was 0.766 (0.746–0.786).

### Prediction of the composite endpoint of myocardial infarction, stroke or death

In total, 70 incident strokes and 38 incident MI occurred. When assessing the quartile groups for time to the composite endpoint of MI, stroke or death (n = 661 without prior events), there were again no differences between the AZGP1 groups in Kaplan–Meier analysis (p = 0.697, Supplementary Fig. S2). Of the 638 cases with complete candidate variables 178 reached the composite endpoint, by counting the first of the events of interest comprising 32 incident MI, 66 incident strokes and 80 deaths. Out of the 20 candidate variables considered for the prediction of the composite endpoint, from 500 bootstrap-based Cox models with backward selection, AZGP1 was among 8 variables included in > 50% of all models (61.0%, Supplementary Table S3). Again, when analyzing the predictive value in the multivariable Cox regression model incorporating the predictive factors (HR; 95%CI) age (1.45; 1.26–1.67), BMI (0.95; 0.91–0.99), waist-hip-ratio (1.31; 1.06–1.62), cystatin C (1.15 per 0.3 mg/l; 1.04–1.28), CRP (1.51 per log(mg/l)-unit; 1.07–2.13) and CCI (1.10; 1.05–1.15), higher AZGP1 levels predicted a lower risk for the composite endpoint (HR per log-unit = 0.43, 0.23–0.82, p = 0.010; Fig. 1B). The c-index of this Cox model was 0.681 (0.683–0.679). The calibration test indicated good model performance (p = 0.498). The model overestimated the observed frequency of the composite endpoint by 1.6–4.1% (Fig. 2B). When using LASSO for variable selection, only age, CCI and cystatin C were selected with HRs which were about half the size of the above described model (Supplementary Table S2). AZGP1 was not included in this model. The corresponding c-index was 0.749 (0.731–0.767).

## Discussion

The purpose of this analysis was to develop a knowledge-based prediction model for mortality and CV events explicitly in a subsample of a large, well-characterized cohort of older adults and to evaluate the predictive potential of the biomarker AZGP1. So far, no valid prediction model for both endpoints in this age group had existed. A proposed prediction model such as the SCORE-model for older people (SCORE-OP) was proven suboptimal when externally validated in the BIS-cohort^24^.

Our analysis identified that, apart from AZGP1, chronological age, BMI, CCI, cystatin C and elevated CRP proved to be significant predictors of both, mortality and the composite CV endpoint. With regard to AZGP1, we found that higher AZGP1 serum levels were predictive for lower mortality and a lower risk for the composite CV endpoint. In the validation step evaluating 500 bootstrap samples AZGP1 was selected into 67% of the models for death, and into 61% of the models for the composite CV endpoint. The finally selected models built on the original subsample both overestimated the observed event-free survival, albeit to a negligible extent, as indicated by the non-significant results of the calibration test. When we performed variable selection with the LASSO method, for the endpoint mortality all 8 variables from the bootstrap-backward-selection (BBS) approach were confirmed with similar coefficients, despite the LASSO model comprising eight additional variables, indicating a small impact of these variables. Given the comparable discriminative ability of both models according to the c-index and aiming at sparse models, we consider the model derived with BBS as preferable. For the composite endpoint the LASSO selection resulted in a small model only comprising age, CCI and cystatin C, to our surprise with a better discrimination index than the BBS model. The three variables selected were also part of the BBS model with 6 variables; regression coefficients for age, CCI and cystatin C in the LASSO model were about half the size of those in the BBS model, presumably since no variables with negative coefficients were part of the LASSO selection, such as BMI and AZGP1 in the BBS model. From a medical perspective the small LASSO model seems rather questionable; to our knowledge the most commonly used risk score in Europe adapted for older people (SCORE-OP) does not perform satisfactorily in older adults^24^. This means there is no straight answer which variables could be missing here to predict cardiovascular risk in the general elder population. Furthermore, it is known that LASSO will select only one feature from a group of correlated features, and the selection is arbitrary in nature^28^. Thus, the surprisingly limited variable selection for the composite cardiovascular endpoint could also be a result of this property.

Although our understanding of the complex molecular changes of aging is increasing, there is still a lack of biomarkers which are able to distinguish between healthy and unhealthy aging. Emerging biomarkers which have been introduced in association with increased CV risk and mortality include markers of inflammation, heart failure or kidney dysfunction, e.g. CRP, Brain Natriuretic Peptide (BNP) and cystatin C^29–33^. In our cohort, we had no information on BNP, but on CRP as well as cystatin C which were both predictive for mortality and the composite endpoint.

Similar to cystatin C, AZGP1 is filtered and degraded by the kidney. Thus, circulating concentrations of AZGP1 also depend on renal function, leading to increased plasma levels in patients with kidney dysfunction^2,3^. In the present study all kidney function parameters (creatinine, cystatin C, eGFR) demonstrated worsening kidney function with increasing levels of AZGP1, though without significant correlation patterns. Higher AZGP1 serum levels predicted reduced outcome risks in contrast to cystatin C levels where higher levels predicted increased outcome risks. In the BIS cohort, a population-based sample of older adults investigating the decline in kidney function, we have previously demonstrated an elevated load of major CV risk factors in individuals with reduced kidney function^25,34^.

While there are no published data on the predictive nature of AZGP1, there are some studies investigating the association of AZGP1 and traditional CV risk factors. Kurita et al., for example, presented a cohort in which higher serum AZGP1 levels were positively associated with hypertension^35^. In contrast, Zhu et al. demonstrated reduced AZGP1 levels in hypertensive patients^9^. A recent cross-sectional study by Huang et al. in middle aged patients (mean age 58 years) indicated an inverse correlation between serum AZGP1 levels and coronary artery disease (CAD)^18^. The authors demonstrated an independent association between low AZGP1 levels and atherosclerosis, and provided experimental evidence indicating that AZGP1 exerts anti-inflammatory effects via the β3-adrenoceptor and JNK/AP-1 signaling in macrophages and possibly in other cells surrounding atherosclerotic plaques. Another study in middle aged patients (mean age 55 years) found a similar association between lower AZGP1 levels and premature CAD^9^. Since the pathophysiologic relationship between AZGP1 and CV risk factors especially in older adults is still unclear, we focused in our analysis on the predictive power of AZGP1 and found AZGP1 to be predictive for mortality and the composite endpoint including myocardial infarction and stroke.

As we investigated a population of older adults, another relevant aspect is the role of AZGP1 in frail individuals. In a previous observational study Lee et al. investigated frailty and AZGP1 levels in participants from a hospital-based comprehensive geriatric assessment program (mean age 77 years)^36^. In contrast to our data they found that AZGP1 levels were higher in men than in women. By multiple linear regression analysis they also observed a positive correlation between AZGP1 and frailty in women, assessed by unintentional weight loss, exhaustion, low physical activity level, slow walking speed and low grip strength. A further comparison of our data with the study by Lee et al. is difficult as we do not have information on frailty parameters. We based our assessment on the CCI which represents the concept of comorbidity and overlaps only partly with the construct of frailty^37^. When investigating individuals of the highest AZGP1 quartile group we found an association with a higher CCI. To minimize the potential influence of impaired kidney function we repeated the analysis without individuals with CKD and found no association between AZGP1 and CCI, suggesting that the increased AZGP-1 levels were due to the presence of CKD and not the higher CCI.

Overall, data on AZGP1 in older adults are scarce. There is only one additional report in which plasma AZGP1 levels were measured in a surgical geriatric patient cohort; Vasunilashorn et al. performed a proteomic study in a subsample of the Successful Aging after Elective Surgery (SAGES) cohort (mean age 77 years)^38^. Interestingly, they found that lower levels of AZGP1 were associated with a higher risk to develop post-surgical delirium in geriatric patients.

Our study has some limitations. First, we cannot draw conclusions about any causative role of AZGP1 levels. It remains unclear whether AZGP1 is actively involved in CV protective mechanisms or whether it should be regarded as a purely predictive biomarker. While there are interesting mechanistic concepts on potential protective functions, we are lacking conclusive evidence about their relevance which is why we chose a predictive and not a causal model. Secondly, AZGP1 measurements were made on stored samples of one time point measurements. This may not reflect AZGP1 dynamics over time. Furthermore, the analyzed subsample was slightly younger and seemingly healthier compared to the overall BIS cohort. Finally, assessment of the discriminatory properties for both prediction models in external data sets is required, as is the evaluation of the predictive potential of AZGP1 in established risk models or proposed risk scores especially developed for older ages. Still, we think that despite the limited model validation and the deviating results of the LASSO variable selection for the composite endpoint, our findings propose that AZGP1 might have potential as a new biomarker for cardiovascular aging. The strengths of our report are based on the size of the database given the advanced mean age of the participants and the comprehensive nature of follow-up data, which provides a well phenotyped sample of community-dwelling older adults. Besides, we applied rigorous prediction modeling methodology. Furthermore, our results contribute a piece to the puzzle of the potential role of AZGP1 in cardiovascular aging.

In conclusion, this is the first study to analyze the predictive potential of AZGP1 serum levels for mortality and CV events in a community-based subsample of older adults in Berlin. Our data revealed that in old age higher serum levels of AZGP1 were predicting a reduced mortality and CV risk within a model of other knowledge-based predictors. Given the challenges of traditional CV risk prediction in older people we suggest that AZGP1 merits further exploration as a novel CV biomarker for healthy aging.

## Methods

### Study design and population

The Berlin Initiative Study (BIS) is a longitudinal population-based cohort of 2069 older adults living in Berlin, who were recruited between November 2009 and June 2011. Inclusion criteria were AOK membership [AOK-Nordost—Die Gesundheitskasse; Berlin’s largest statutory health insurance fund] and age of 70 years and above. Follow-up information was collected bi-annually. A detailed description of the study design can be found elsewhere^26^. Additionally, insurance claims data including “International Classification of Diseases”-10 (ICD-10) codes^39^ were available for all study participants. The Charlson comorbidity index (CCI) was calculated based on ICD-10 codes, and the age-adjusted CCI version was used^40^. The study was approved by the local ethics committee, Charité, Berlin, Germany (EA2/009/08), and every participant gave written informed consent. All research was performed in accordance with relevant guidelines and regulations and the Declaration of Helsinki. The data base freeze for this analysis was December 04, 2019.

Data for the current study include a subsample of 930 out of 1440 BIS study participants who attended the 4-year follow up visit of the BIS between January 2014 and September 2015. Due to a change of the laboratory analysis platform provider during the 4-year follow up visit, reserve blood samples had been stored for the first 65% of participants until June 2015. Those 930 samples were available for the additional laboratory analyses of AZGP1 conducted in 2018 (Supplementary Fig. S1).

### Laboratory methods

AZGP1 (μg/ml) was measured in frozen serum samples (− 80 C) in a subsample of 930 individuals of the 4-year follow-up visit using a previously described^2^ commercial enzyme-linked immunosorbent assay (Biovendor, Modrice, Czech Republic), according to the manufacturer’s instructions. Investigators were blinded to patients’ data and all measurements were performed in duplicate in 2018. The assay sensitivity was 0.673 ng/ml. The intra-assay coefficient of variation was less than 5%. Serum creatinine was analysed using the isotope dilution mass spectrometry (IDMS) traceable enzymatic method from Roche (Crea plus; Roche Diagnostics, Mannheim, Germany) on a Roche Modular-analyzer P-Modul. Cystatin C was measured using the particle enhanced turbidimetric (PETIA) Tina-quant generation 2 assay on the Roche/Hitachi Cobas S system (Cobas c 501).

The estimated glomerular filtration rate (eGFR) was calculated with the combined creatinine and cystatin C-based BIS2 equation (eGFR_BIS2_) developed for individuals above the age of 70^41,42^.

### Statistical analysis

The biomarker AZGP1 was log(10)-transformed for further analyses. To recognize potential trends in relation to demographic and clinical characteristics with rising AGZP1 serum levels, the cohort was split into AGZP1 quartile groups. Correlations of AZGP1 with continuous variables were evaluated with Pearson and Spearman correlation coefficients, for categorical variables biomarker levels were compared with the t-test. We investigated the predictive value of serum AZGP1 levels for the outcome mortality and the composite endpoint comprising the outcomes stroke, myocardial infarction (MI) or death, whatever occurred first. Information on mortality, acute and prior events of MI (ICD10: I21, only prior I22, I23, I25.2) and stroke (ICD10: I61, I63, I64, only prior I69.1, I69.2, I69.3, I69.4) were derived from the claims data. Individuals with a previous event of the composite endpoint (stroke or MI) by insurance claims data before the AZGP1 measurement (n = 269) were excluded from the respective analyses (ICD-10 codes from insurance claims data reach back to 2006).

For time-to-event analysis we used Kaplan–Meier analysis with the Breslow test by quartile groups of the biomarkers, and Cox proportional hazard models with stepwise backward selection. Based on medical pre-selection, the following variables were considered for the predictive models: age, gender, body-mass-index (BMI), waist-to-hip-ratio, smoking, anemia, systolic and diastolic blood pressure, serum creatinine, serum cystatin C, hemoglobin, high density lipoprotein (HDL), low density lipoprotein (LDL) and total cholesterol, C-reactive protein (CRP, log-transformed), eGFR_BIS2_, albumin-to-creatinine ratio (ACR, log-transformed), age-corrected Charlson Comorbidity Index (CCI), antihypertensive treatment, and AZGP1 serum levels (log-transformed). Further comorbidities such as diabetes mellitus, myocardial infarction, stroke, cancer and coronary heart disease were included as parts of the CCI. To illustrate the fit of these models, calibration plots are shown comparing survival estimates with 95% confidence interval from the Cox models to the Kaplan–Meier survival rates; the modified D'Agostino-Nam test on calibration of the Cox model was performed^43,44^. To assess model discrimination, a generalized version of the c-index for survival analysis allowing for censoring was calculated^45^. For internal model validation, 500 bootstrap samples were drawn from the subsample and Cox regression with backward selection was applied. The mean of the differences in c-indices of the corresponding models between the bootstrap and the original subsample was calculated as indicator for the optimism in the c-index of the prediction model derived from the original subsample^46^. The c-indices corrected for optimism are reported. Furthermore, to check the robustness of our model results with other statistical selection techniques, we additionally performed the LASSO method for Cox regression^47^ (package glmnet in R^48^) in the original data. For comparison, we show the variable selection from LASSO when choosing λ where the cross-validation error curve hits its minimum; c-indices from the incorporated tenfold cross-validation function are reported.

Since information on death and endpoints derived from claims data are only available with a respective time lag, analyses of death were censored at 30.06.2019, and analyses for the composite endpoint were censored at 31.12.2018. We conducted complete-case analyses. IBM SPSS Statistics version 25.0^49^ (https://www.ibm.com/de-de/analytics/spss-statistics-software) and R version 4.0.0^48^ (https://www.R-project.org) were used for analysis. We followed the TRIPOD reporting guidelines^50^.

## Supplementary Information

Supplementary Information.

## Data Availability

The datasets generated during and/or analysed during the current study are not publicly available due to restrictions in the patient consent form but are available from the corresponding author on reasonable request.
