# Supplementary material for: Development of a prediction model for mortality and cardiovascular outcomes in older adults taking into account AZGP1
Source: Sci Rep. 2021 Jun 3;11:11792. doi: 10.1038/s41598-021-91169-4 (PMC8175433; doi:10.1038/s41598-021-91169-4)

Development of a prediction model for mortality and cardiovascular outcomes in older adults taking into account AZGP1

Dörte Huscher; Natalie Ebert; Inga Soerensen-Zender; Nina Mielke; Elke Schaeffner* and Roland Schmitt*

Supplementary Tables and Figures

Supplementary Table S1: Baseline characteristics at 4 year follow-up in patients with and without AZGP1 measurement

Supplementary Table S2: Stepwise backward selection of variables in Cox regression analysis for the outcome death based on 500 bootstrap samples with replacement and results from the LASSO variable selection in Cox regression

Supplementary Table S3: Stepwise backward selection of variables in Cox regression analysis for the outcome composite endpoint based on 500 bootstrap samples with replacement and results from the LASSO variable selection in Cox regression

Supplementary Figure S1: Flow chart to AZGP1 subsample of BIS cohort. The figure was produced with MS Office Powerpoint 2016 (https://www.microsoft.com/de-de/microsoft-365).

Supplementary Figure S2: Kaplan-Meier curves of AZGP1 quartile groups split by $eGFR_{BIS2}$ (ml/min/1.73 m²) for A) time to death and B) time to composite endpoint; at the bottom of each graph case numbers at risk are shown. The figure was produced with IBM SPSS Statistics version 25.0 (https://www.ibm.com/de-de/analytics/spss-statistics-software) and MS Office Powerpoint 2016 (https://www.microsoft.com/de-de/microsoft-365).

Supplementary Table S1: Baseline characteristics at 4-year follow-up in patients with and without AZGP1 measurement

	**Cohort**	**with AZGP1**	**without AZGP1**
N (% of cohort)	1440	930 (64.6)	510 (35.4)
Age (years), mean±SD	83.1 ± 6.1	82.3 ± 5.6	84.7 ± 6.7
Female, n (%)	726 (50.4)	439 (47.2)	287 (56.3)
Smoking ever, n (%)	684 (47.5)	460 (49.5)	224 (43.9)
Body mass index (kg/m²), mean±SD	27.5 ± 4.3	27.8 ± 4.4	27.1 ± 4.3
Body mass index (kg/m²), n (%)	1423	922	501
< 25	412 (29.0)	249 (27.0)	163 (32.5)
25-< 30	663 (46.6)	439 (47.6)	224 (44.7)
≥ 30	348 (24.5)	234 (25.4)	114 (22.8)
Waist to hip ratio, mean±SD	0.92 ± 0.08	0.92 ± 0.08	0.92 ± 0.09
Waist to hip ratio, n (%)	1409	916	493
normal weight (female<0.8, male<0.9)	143 (10.1)	92 (10.0)	51 (10.3)
overweight (female 0.8-<0.85, male 0.9-<1.0)	530 (37.6)	351 (38.3)	179 (36.3)
adipositas (female>=0.85, male>=1.0)	736 (52.2)	473 (51.6)	263 (53.3)
Diabetes mellitus, n (%)	347/1413 (24.6)	215/921 (23.3)	132/492 (26.8)

Myocardial infarction#, n (%)	256/1432 (17.9)	157/922 (17.0)	99/510 (19.4)
Stroke#, n (%)	237/1432 (16.6)	149/922 (16.2)	55/510 (17.3)
Heart failure, n (%)	56/1435 (3.9)	36/927 (3.9)	20/508 (3.9)
Cancer#, n (%)	485/1432 (33.9)	323/922 (35.0)	162/510 (31.8)
Anemia, n (%)	253/1412 (17.9)	161/922 (17.5)	92/490 (18.8)
Charlson comorbidity index, n (%)	1431	921	510
3-4	106 (7.4)	75 (8.1)	31 (6.1)
5-7	385 (26.9)	264 (28.7)	121 (23.7)
8-10	446 (31.2)	279 (30.3)	167 (32.7)
≥11	494 (34.5)	303 (32.9)	191 (37.5)
Systolic blood pressure (mmHg), mean±SD	143.5 ± 21.4	143.3 ± 21.7	144.0 ± 21.0
Hemoglobin (g/dl)	13.5 ± 1.4	13.5 ± 1.4	13.5 ± 1.5
Cholesterol level (mg/dL), mean±SD	211.5 ± 49.6	213.7 ± 50.2	207.3 ± 48.0
LDL cholesterol (mg/dL), mean±SD	123.5 ± 41.8	123.1 ± 42.3	124.1 ± 40.8
HDL cholesterol (mg/dL), mean±SD	60.7 ± 19.2	62.0 ± 19.5	58.4 ± 18.5
CRP (mg/dl), median (IQR)	1.6 (0.8;3.4)	1.6 (0.8;3.3)	1.9 (0.9;3.5)
Creatinine - serum (mg/dl), mean±SD	1.05 ± 0.41	1.04 ± 0.39	1.08 ± 0.45
Cystatin C (mg/l)	1.35 ± 0.48	1.35 ± 0.45	1.35 ± 0.54
$eGFR_{BIS2}$ (ml/min/1.73 m^2), mean±SD	51 ± 14	52 ± 13	51 ± 14

eGFR $_{BIS2}$<60, n (%)	1004/1396 (71.9)	674/926 (72.8)	330/470 (70.2)
ACR (mg/g), median (IQR)	12.0 (4.6;36.6)	11.3 (4.7;33.9)	13.9 (3.5;40.0)
ACR (mg/g), n (%)	1411	916	495
≤30	998 (70.7)	664 (72.5)	334 (67.5)
>30-300	357 (25.3)	219 (23.9)	138 (27.9)
>300	56 (4.0)	33 (3.6)	23 (4.6)

Myocardial infarction, stroke and cancer were derived from insurance claims data based on ICD-10 codes. ACR: albumin creatinine ratio; CRP: c-reactive protein; eGFR: estimated glomerular filtration rate; HDL: high-density lipoprotein; LDL: low-density lipoprotein.

Supplementary Table S2: Stepwise backward selection of variables in Cox regression analysis for the outcome death based on 500 bootstrap samples with replacement; variables are sorted by descending frequency of selection into multivariable model, the total number (n) and proportion of the 500 models (%) is shown; the third column shows regression coefficients of the final stepwise-backward (SB) model in the original data. The last column shows regression coefficients of the LASSO model in the original data, when choosing λ where the cross-validation error curve hits its minimum.

	Selection into multivariable model		Regression coefficient B (SB)	Regression coefficient B (LASSO)
	n	%		
Age, per 5 years	500	100.0	0.475	0.458
Charlson comorbidity index	498	99.6	0.100	0.088
BMI	496	99.2	-0.087	-0.087
Cystatin C, per 0.3 mg/l	486	97.2	0.234	0.228
Smoking ever	363	72.6	0.444	0.320
log(AZGP1)	337	67.4	-0.817	-0.606
Anemia	311	62.2	0.364	0.345
log(CRP)	276	55.2	0.375	0.269
log(ACR)	248	49.6	-	0.165
LDL cholesterol (mg/dL)	240	48.0	-	0.002
Waist to hip ratio, per 0.1 units	208	41.6	-	0.130
Systolic blood pressure (mmHg)	207	41.4	-	-0.005
$eGFR_{BIS2}$ (ml/min/1.73 m^2)	151	30.2	-	-
Cholesterol level (mg/dL)	147	29.4	-	-
HDL cholesterol (mg/dL)	138	27.6	-	-0.005
Creatinine – serum, per 0.3 mg/dl	100	20.0	-	-0.034
Diastolic blood pressure (mmHg)	84	16.8	-	-0.001
Male sex	82	16.4	-	0.079
Hemoglobin (g/dl)	82	16.4	-	-
Antihypertensive medication	38	7.6	-	-

ACR, albumin creatinine ratio; BMI, body mass index; CRP, c-reactive protein; eGFR, estimated glomerular filtration rate; HDL- high-density lipoprotein; LDL- low-density lipoprotein.

Supplementary Table S3: Stepwise backward selection of variables in Cox regression analysis for the outcome composite endpoint based on 500 bootstrap samples with replacement; variables are sorted by descending frequency of selection into multivariable model, the total number (n) and proportion of the 500 models (%) is shown; the third column shows regression coefficients of the final stepwise-backward (SB) model in the original data. The last column shows regression coefficients of the LASSO model in the original data, when choosing λ where the cross-validation error curve hits its minimum.

	Selection into multivariable model		Regression coefficient B (SB)	Regression coefficient B (LASSO)
	n	%		
Age, per 5 years	497	99.4	0.370	0.226
Charlson comorbidity index	462	92.4	0.094	0.051
Waist to hip ratio, per 0.1 units	388	77.6	0.272	-
BMI	376	75.2	-0.056	-
Cystatin C, per 0.3 mg/l	321	64.2	0.141	0.073
log(AZGP1)	305	61.0	-0.843	-
log(CRP)	255	51.0	0.411	-
Creatinine – serum, per 0.3 mg/dl	211	42.2	-	-
Diastolic blood pressure (mmHg)	152	30.4	-	-
Systolic blood pressure (mmHg)	144	28.8	-	-
log(ACR)	125	25.0	-	-
Smoking ever	120	24.0	-	-
$eGFR_{BIS2}$ (ml/min/1.73 m²)	115	23.0	-	-
Male sex	105	21.0	-	-
Anemia	74	14.8	-	-
Cholesterol level (mg/dL)	65	13.0	-	-
LDL cholesterol (mg/dL)	61	12.2	-	-
HDL cholesterol (mg/dL)	61	12.2	-	-
Hemoglobin (g/dl)	53	10.6	-	-
Antihypertensive medication	31	6.2	-	-

ACR: albumin creatinine ratio; BMI: body mass index; CRP: C-reactive protein; eGFR: estimated glomerular filtration rate; HDL: high-density lipoprotein; LDL: low-density lipoprotein.

Supplementary Figure S1: Flow chart to AZGP1 subsample of BIS cohort. The figure was produced with MS Office Powerpoint 2016 (https://www.microsoft.com/de-de/microsoft-365).

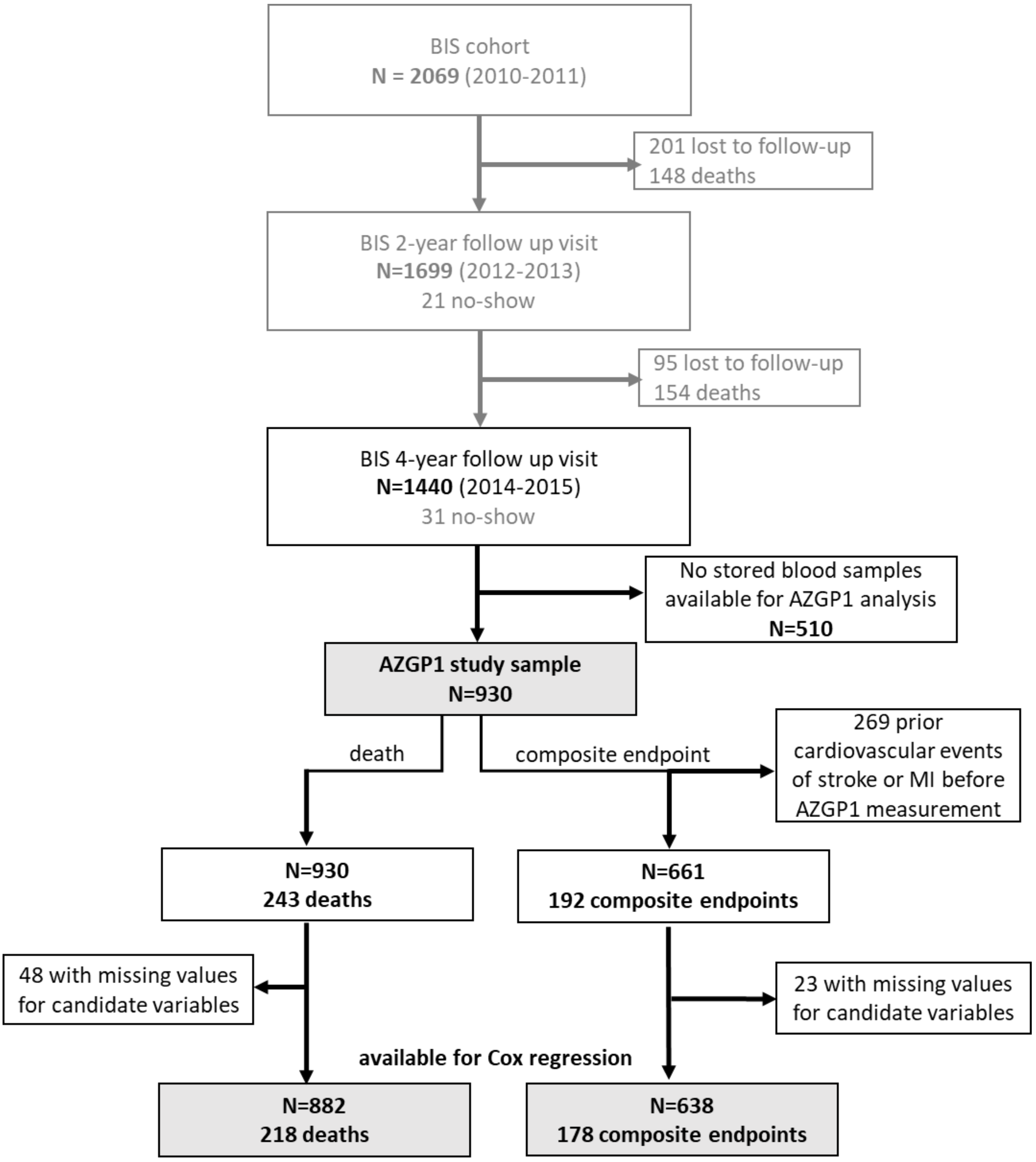

Supplementary Figure S2: Kaplan-Meier curves of AZGP1 quartile groups split by eGFR$_{BIS2}$ (ml/min/1.73 m²) for A) time to death and B) time to composite endpoint; at the bottom of each graph case numbers at risk are shown. The figure was produced with IBM SPSS Statistics version 25.0 (https://www.ibm.com/de-de/analytics/spss-statistics-software) and MS Office Powerpoint 2016 (https://www.microsoft.com/de-de/microsoft-365).

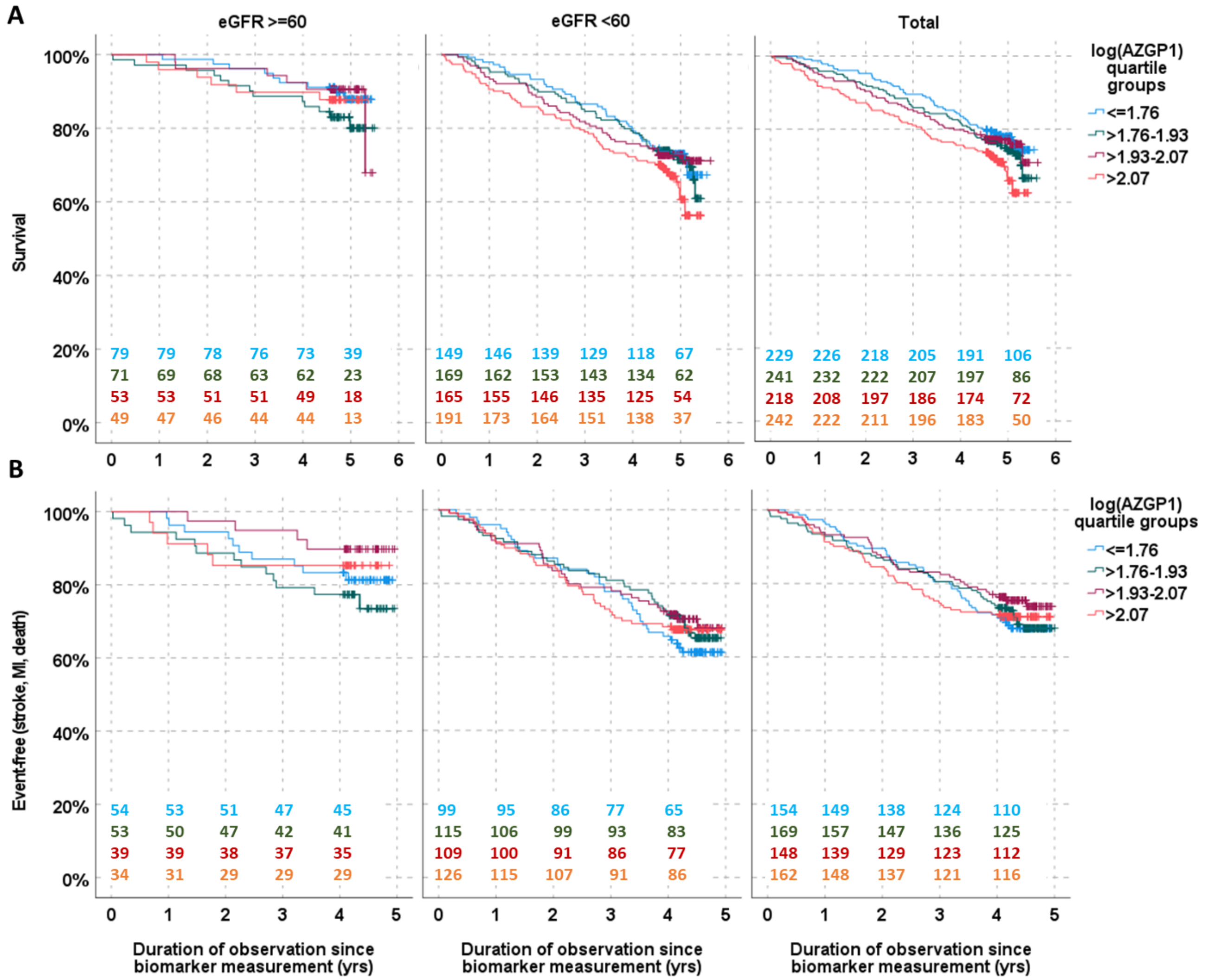

Supplement: Supplementary file 1 — Supplementary Information. [file 41598_2021_91169_MOESM1_ESM.pdf]
